# Exosomes derived from HTLV-1 infected cells contain the viral protein Tax

**DOI:** 10.1186/1742-4690-11-S1-O46

**Published:** 2014-01-07

**Authors:** Aarthi Narayanan, Elizabeth Jaworski, Rachel Van Duyne, Sergey Iordanskiy, Irene Guendel, Ravi Das, Robert Currer, Gavin Sampey, Myung Chung, Kylene Kehn-Hall, Charles Bailey, Anastas Popratiloff, Fatah Kashanchi

**Affiliations:** 1 Department of Molecular and Microbiology, National Center for Biodefense & Infectious Diseases, George Mason University, Manassas, VA, USA; 2 Department of Microbiology, Immunology, and Tropical Medicine, The George Washington University Medical Center, Washington, DC, USA; 3 Center for Microscopy and Image Analysis, The George Washington University Medical Center, Washington, DC, USA

## 

Human T-lymphotropic virus type 1 (HTLV-1) is the causative agent of adult T-cell leukemia. The HTLV-1 transactivator protein Tax has been identified as a critical component in the proliferation and transformation of human primary T-cells. This 40 kDa phosphoprotein not only manipulates chromatin remodeling within the host, but also subverts host cell DNA damage response mechanisms, cell cycle progression, and apoptosis. Here we utilized a combination of filtration and ultracentrifugation methods to enrich for exosomes from culture supernatants of HTLV-1 infected cells. We then employed western blots, mass spectrometry, and cytokine arrays to proteomically characterize the host and viral components in these exosomes. Additionally, RT-PCR was used to determine the presence of viral transcripts in these exosomes. Our results demonstrate that exosomes derived from HTLV-1 infected cells contain traditional exosome proteins. Furthermore, our proteomics studies revealed that these exosomes contain viral components such as gp46 and Tax, as well as inflammatory mediators including IL-6 and IL-10. We also investigated the presence of HTLV-1 mRNA transcripts of Env, Tax, HBZ, and 5’LTR contained within exosomes. Moreover, we evaluated the functional impacts of treating naïve recipient cells with exosomes secreted from HTLV-1 infected cells, and determined that the exosomes were able to induce a response in reactive oxygen species production. Our observation that the viral protein Tax is contained within exosomes and may be transmitted in an extracellular capacity raises important implications to pathogenesis associated with HTLV-1 infections.

